# Supplementary material for: Computational identification of mutually exclusive transcriptional drivers dysregulating metastatic microRNAs in prostate cancer
Source: Nat Commun. 2017 Apr 11;8:14917. doi: 10.1038/ncomms14917 (PMC5394245; doi:10.1038/ncomms14917)
Supplement: Supplementary Information — Supplementary figures, supplementary tables and supplementary references. [file ncomms14917-s1.pdf]

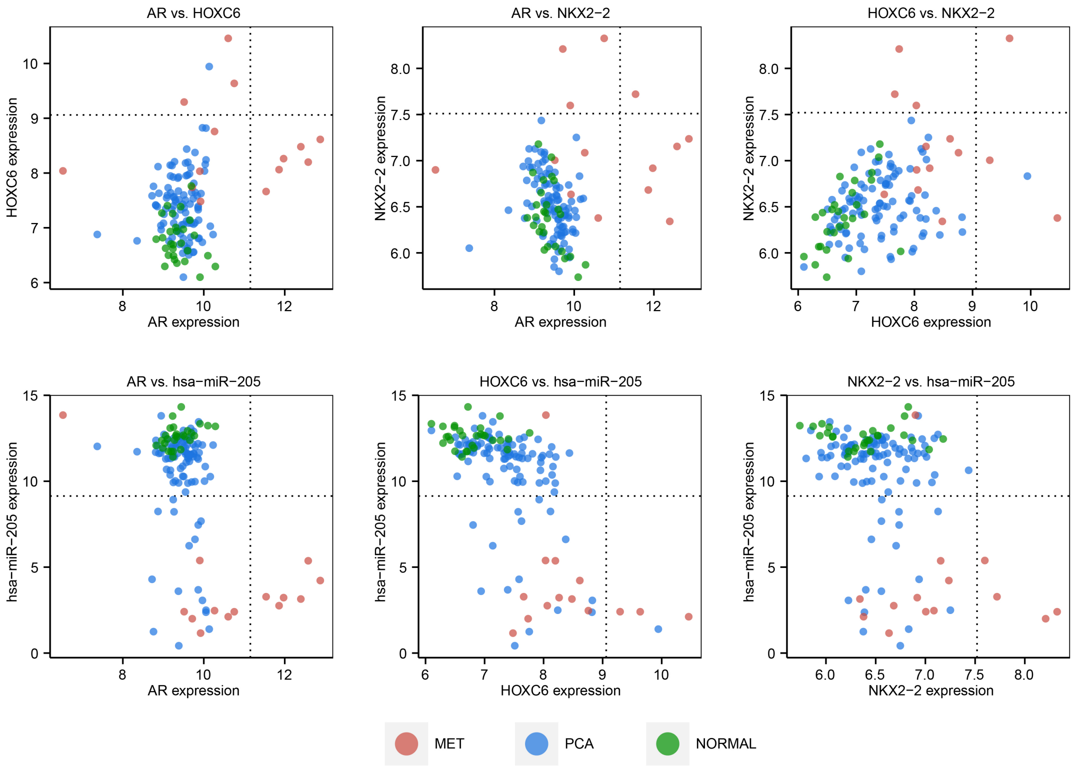

Supplementary Figure 1. Example expression patterns of mutually exclusive driver TFs and target microRNAs.

Top panels showing the mutually exclusive relationship between the overexpressed outliers of *AR*, *HOXC6* and *NKX2-2*. Bottom panels showing the significant association between the three TFs' overexpressed outliers with a putative target microRNA's underexpressed outliers. Dotted lines represent the cutoffs for outliers.

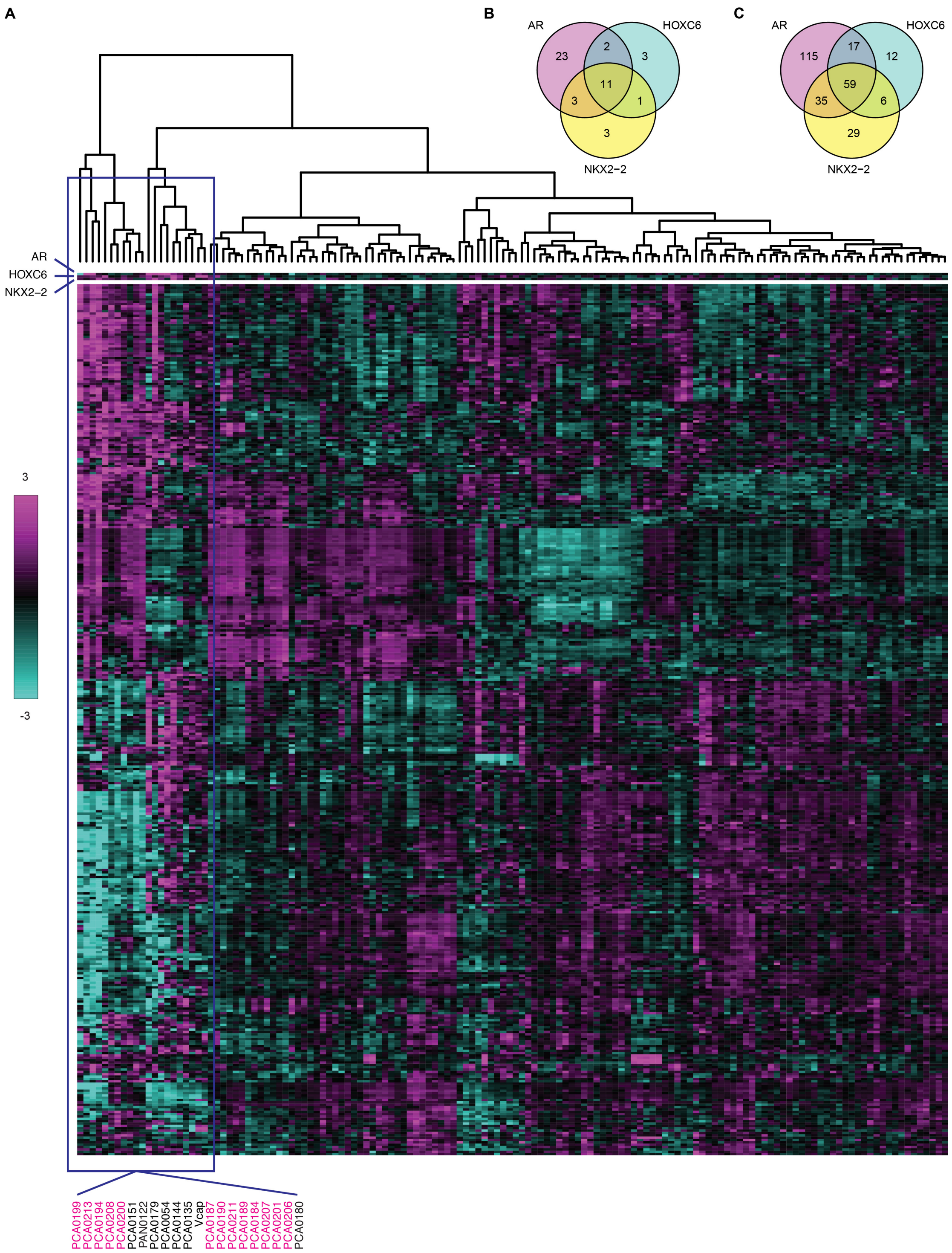

Supplementary Figure 2. Computational prediction showing that AR, HOXC6 and NKX2-2 co-regulate the same set of microRNAs.

(A) Heatmap showing the clustering results using expression profiles of *AR*, *HOXC6*, *NKX2-2* and all microRNAs. Black box denotes the cluster enriched with metastatic prostate cancers. The names for all samples in the metastasis cluster are listed and metastatic ones are colored in red. (B) Venn diagram showing the overlap between predicted microRNA targets for *AR*, *HOXC6* and *NKX2-2*. (C) Venn diagram showing the overlap among microRNAs regulated by *AR*, *HOXC6* and *NKX2-2* based on the miRNA-seq data.

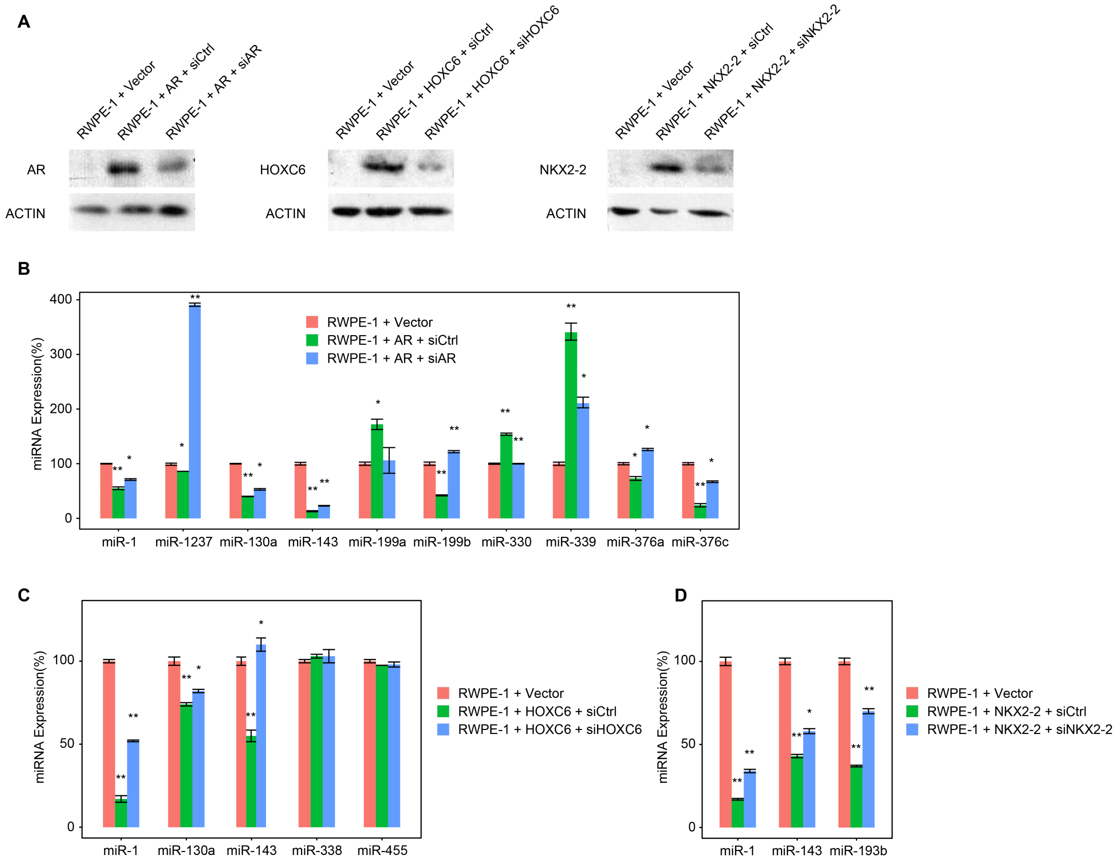

E

	AR	HOXC6	NKX2-2
Number of miRs validated	18	7	11
Number of miRs not validated	17	6	4
Rate of validation	51%	54%	73%

Supplementary Figure 3. Experimental validation of selected predictions.

(A) Immunoblotting analysis showing specific siRNAs for *AR*, *HOXC6* or *NKX2-2* reducing the target protein's expression. (B) Bar charts showing qRT-PCR results for selected microRNAs predicted to be regulated by *AR*. n = 3; error bars indicate mean ± s.d. * $p < 0.05$; ** $p < 0.01$, as determined using the two-tailed Student's t-test. (C) Same as (B) for *HOXC6* regulated microRNAs. (D) Same as (B) for *NKX2-2* regulated microRNAs. (E) High rates of validation of computationally predicted microRNA targets by miRNA-seq and qRT-PCR. Mature microRNAs derived from -3p or -5p of the same microRNA precursors were counted as one. Results shown are representative of three independent experiments.

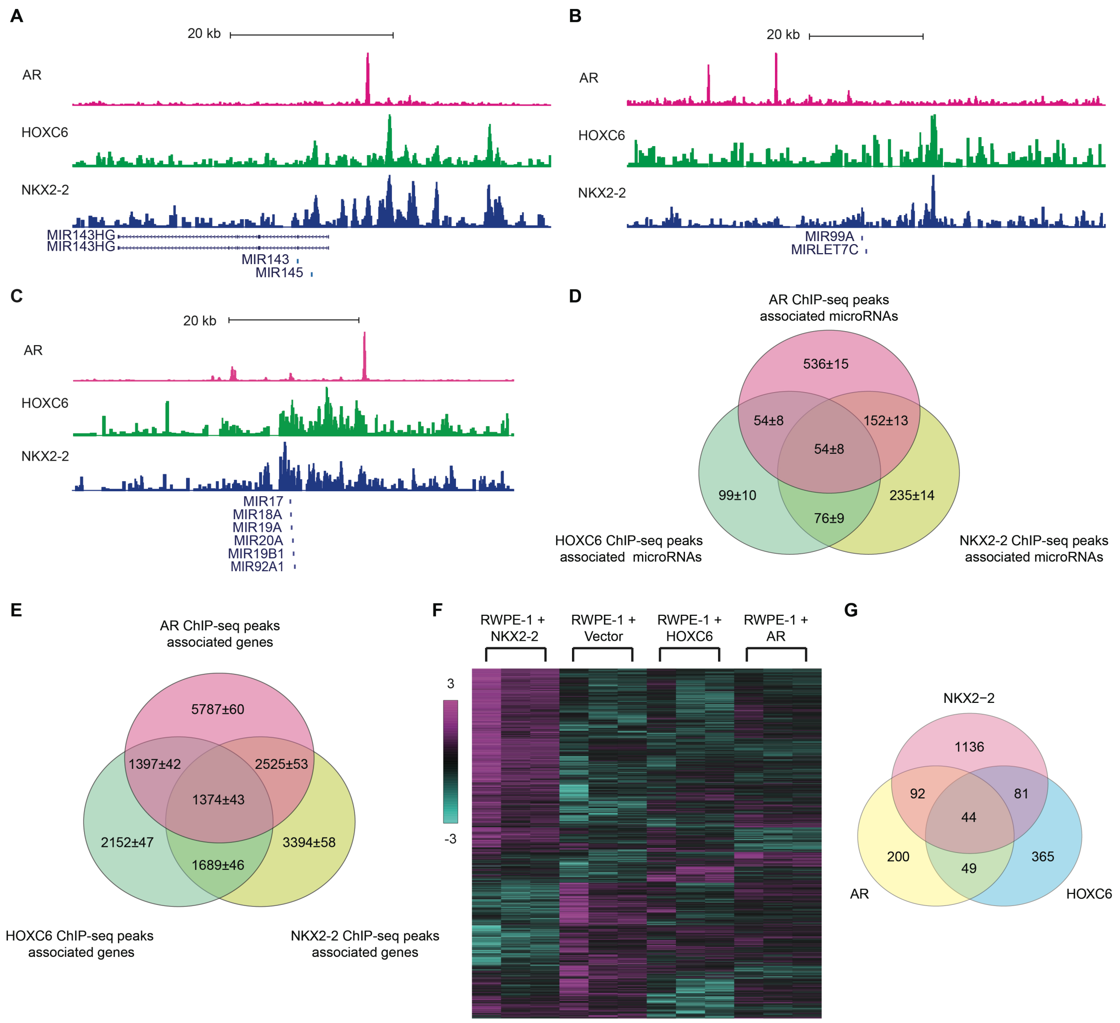

Supplementary Figure 4. Public ChIP-seq data supports that AR, HOXC6 and NKX2-2 regulate a common set of microRNAs.

(A) Genome browser representation of *AR*, *HOXC6* and *NKX2-2* binding events associated miR-143 and miR-145. (B) Same as (A) for miR-99a and Let-7c. (C) Same as (A) for miR-17-92 cluster. (D) Venn diagram showing the overlap of the microRNAs associated with *AR*, *HOXC6* and *NKX2-2* binding events derived via bootstrapping of published ChIP-seq data. (E) Venn diagram showing the overlap of coding genes associated with *AR*, *HOXC6* and *NKX2-2* binding events derived via bootstrapping of published ChIP-seq data. (F) Heatmap of mRNA microarray results showing differentially expressed mRNAs regulated by *AR*, *HOXC6* or *NKX2-2*. (G) Venn diagram showing the overlap of the mRNAs regulated by *AR*, *HOXC6* or *NKX2-2* derived from mRNA microarray analysis.

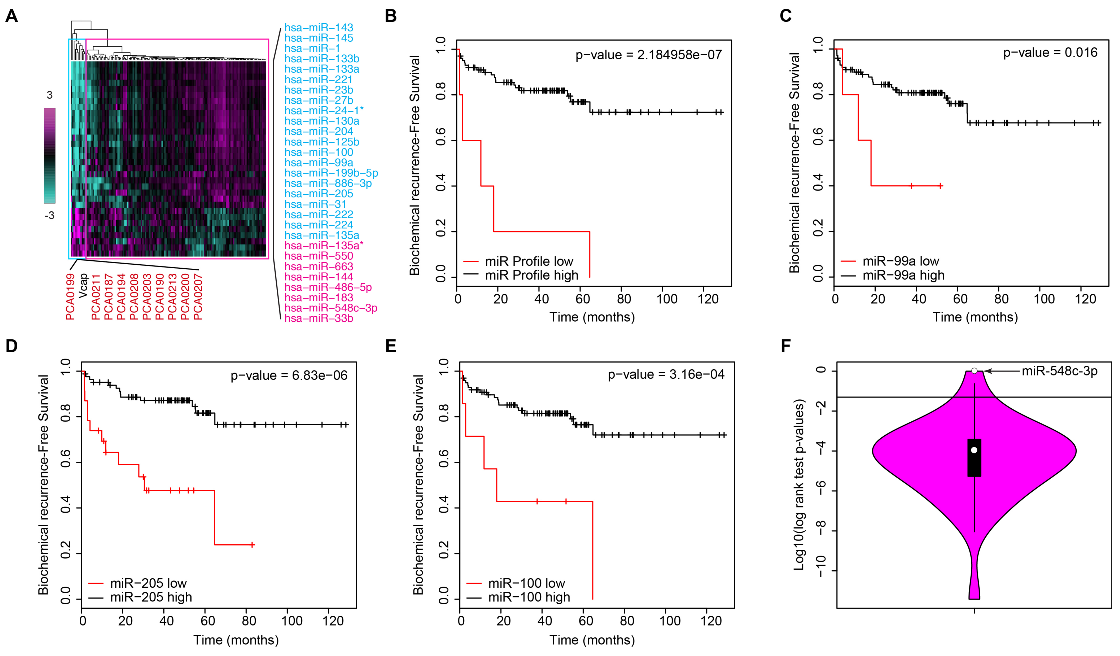

Supplementary Figure 5. MicroRNAs differentially expressed in metastatic prostate cancer are of significant prognostic values.

(A) Heatmap showing the clustering results using the expression profile of all microRNAs differentially expressed in metastatic prostate cancer. Cyan box denotes the cluster enriched with metastatic prostate cancer samples. The names for all samples in the metastasis cluster are listed and metastatic ones are colored in red. Magenta names indicate microRNAs that were upregulated in metastatic prostate cancer and cyan names indicate microRNAs that were downregulated in metastatic prostate cancer. (B-E) Kaplan-Meier survival analysis based on the expression profile of all metastasis-associated microRNAs (B), miR-99a (C), miR-205 (D) and miR-100 (E). (F) Violin plot summarizing Log rank test p-values derived from survival analyses using each of the metastasis-associated microRNAs.

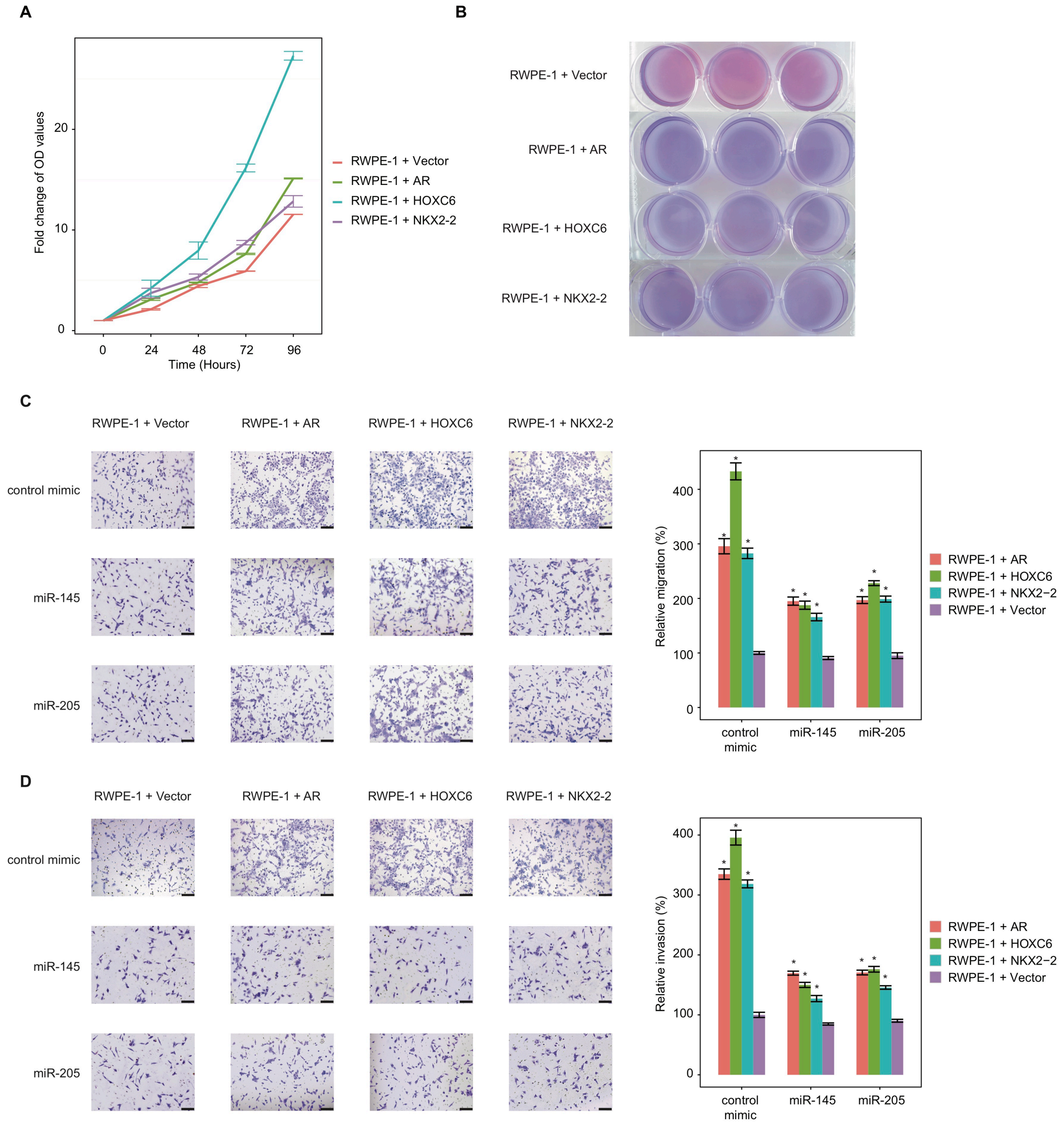

Supplementary Figure 6. Functional validation of AR, HOXC6 and NKX2-2.

(A) Result of MTS assay. (B) Representative images showing the results of colony formation assay. (C) Representative images showing the results of the migration assay. Pictures in the top row are RWPE-1 cells overexpressing *AR*, *HOXC6*, *NKX2-2* or a control empty vector treated with control microRNA mimics. Pictures in the middle row are cells treated with miR-145 mimics. Pictures in the bottom row are cells treated with miR-205 mimics. Scale bars: 100 μm. The number of migrated cells were quantified and illustrated in the bar chart. * $p < 0.01$, as determined using the two-tailed Student's t-test. (D) Same as (C) for the invasion assay. Results shown are representative of three independent experiments.

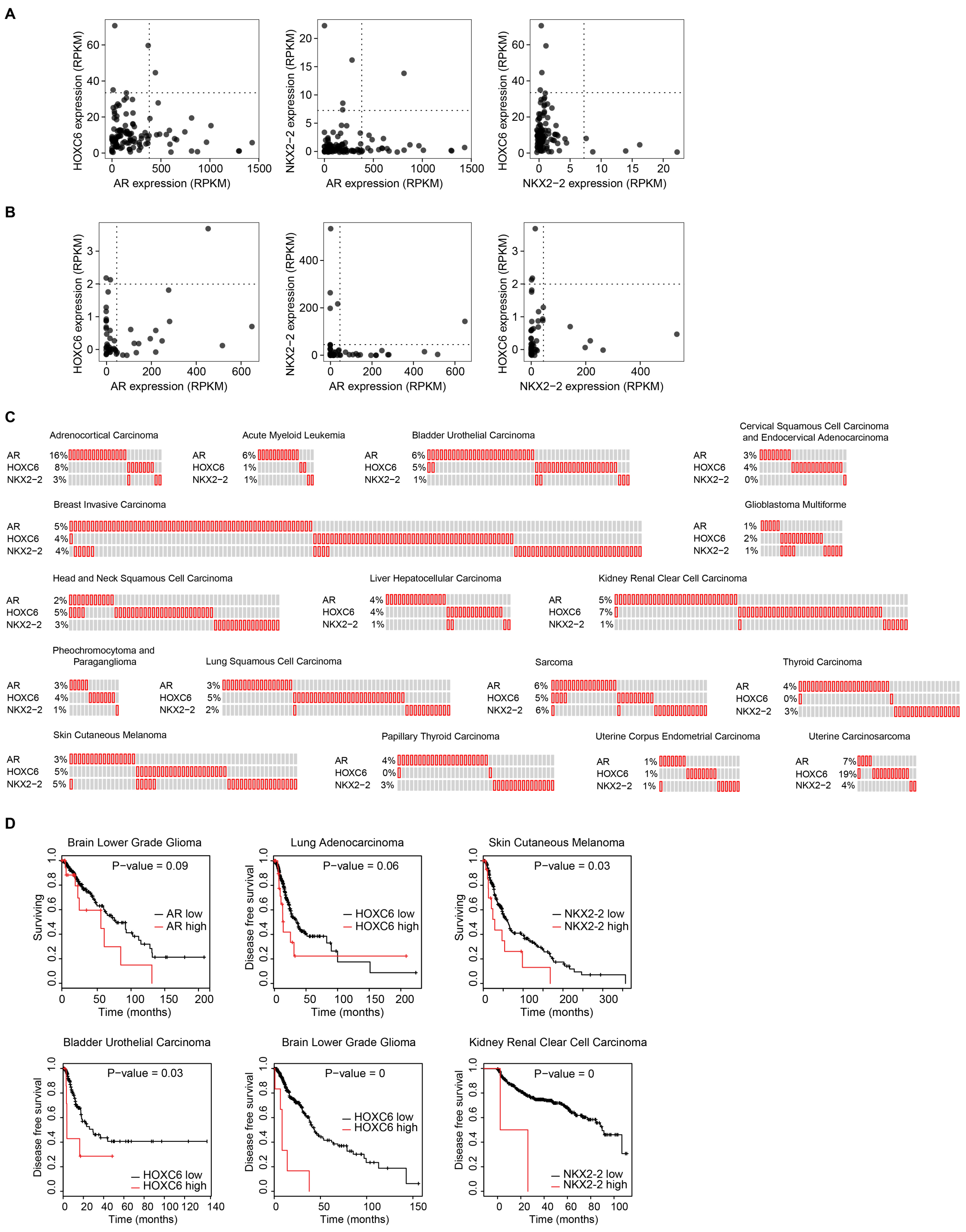

Supplementary Figure 7. The mutually exclusive overexpression of AR, HOXC6 and NKX2-2 was preserved across a large collection of cancer data sets.

(A) Scatter plots showing the mutually exclusive overexpression of *AR*, *HOXC6* and *NKX2-2* using the Robinson data set for CRPC. Dotted lines represent cutoffs for overexpression. (B) Same as (A) for the Beltran data set. (C) Oncoprints showing mutually exclusive overexpression of *AR*, *HOXC6* and *NKX2-2* in 17 TCGA cancers. (D) Kaplan-Meier survival analyses based on the overexpression of *AR*, *HOXC6* or *NKX2-2* in different TCGA cancer data sets.

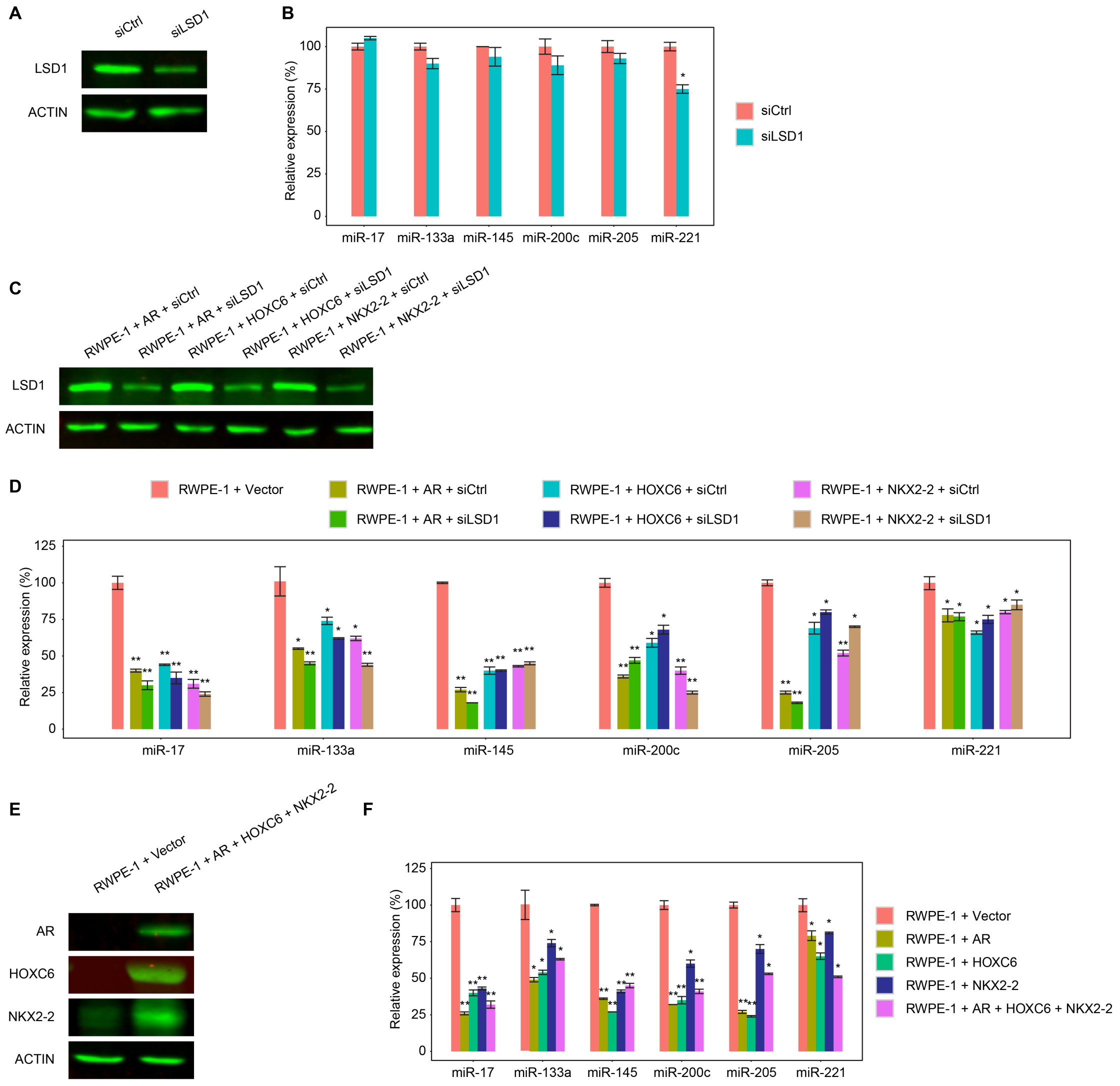

Supplementary Figure 8. LSD1 knockdown and TF co-overexpression didn't impact the expression of metastasis-associated miRNAs.

(A) Immunoblotting analysis showing specific siRNAs for *LSD1* reducing the target protein's expression in LnCAP cells. (B) Bar charts showing qRT-PCR results for selected microRNAs after *LSD1* knockdown in LnCAP cells. n = 3; error bars indicate mean ± s.d. * $p < 0.05$; ** $p < 0.01$, as determined using the two-tailed Student's t-test. (C) Immunoblotting analysis showing specific siRNAs for *LSD1* reducing the target protein's expression in RWPE-1 cells overexpressing *AR*, *HOXC6* or *NKX2-2*. (D) Bar charts showing qRT-PCR results for selected microRNAs after *LSD1* knockdown in RWPE-1 cell overexpressing *AR*, *HOXC6* or *NKX2-2*. (E) Immunoblotting analysis showing target protein abundance in RWPE-1 cells simultaneously overexpressing *AR*, *HOXC6* and *NKX2-2*. (F) Bar charts showing qRT-PCR results for selected microRNAs in RWPE-1 cell simultaneously overexpressing *AR*, *HOXC6* and *NKX2-2*. Results shown are representative of three independent experiments.

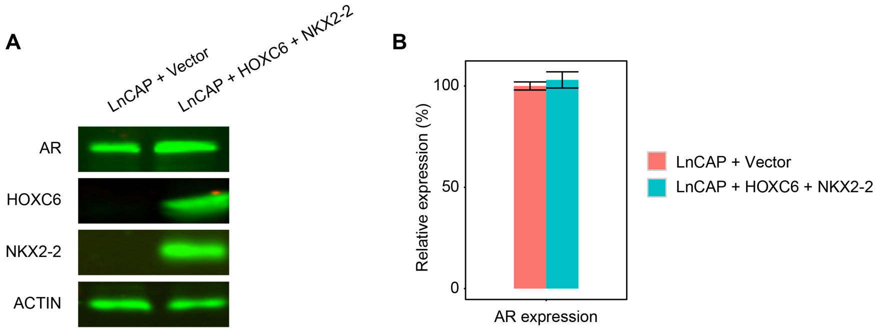

Supplementary Figure 9. HOXC6 and NKX2-2 doesn't impact AR expression.

(A) Immunoblotting analysis showing target protein abundance in LnCAP cells overexpressing *HOXC6* and *NKX2-2* or a control empty vector. (B) Bar plots showing the level of *AR* mRNA. Results shown are representative of three independent experiments.

Supplementary Table 1. Experimental validation of computationally predicted microRNA targets of AR, HOXC6 and NKX2-2.

TF	microRNA	Validated	Validation method	Fold change	FDR
AR	hsa-miR-1	Y	qPCR (Fig. S3B)		
	hsa-miR-100	N*	miR-seq	-1.02	9.05E-05
	hsa-miR-1237	N*	qPCR (Fig. S3B)		
	hsa-miR-125b	Y	miR-seq	3.02	3.51E-17
	hsa-miR-130a	Y	qPCR (Fig. S3B)		
	hsa-miR-133a	Y	qPCR (Fig. 2C)		
	hsa-miR-133b	N	miR-seq	NA	NA
	hsa-miR-136*	Y	miR-seq	2.15	0.036
	hsa-miR-143	Y	qPCR (Fig. S3B)		
	hsa-miR-145	Y	qPCR (Fig. 2C)		
	hsa-miR-152	N*	miR-seq	-1.37	2.79E-03
	hsa-miR-15a*	N*	miR-seq	0.70	0.015
	hsa-miR-194*	Y	miR-seq	-1.09	3.83E-05
	hsa-miR-199a-5p	N*	qPCR (Fig. S3B)		
	hsa-miR-199b-5p	Y	qPCR (Fig. S3B)		
	hsa-miR-200c*	N*	qPCR (Fig. 2C)		
	hsa-miR-205	Y	qPCR (Fig. 2C)		
	hsa-miR-218	N	miR-seq	0.097	0.98
	hsa-miR-221	Y	qPCR (Fig. 2C)		
	hsa-miR-222	N	miR-seq	-0.53	0.44
	hsa-miR-23b	N*	miR-seq	-1.46	2.67E-04
	hsa-miR-24	Y	miR-seq	0.61	9.18E-03
	hsa-miR-24-1*	N	miR-seq	-0.59	0.30
	hsa-miR-27a	Y	miR-seq	0.63	0.032
	hsa-miR-27b	N*	miR-seq	-0.92	1.53E-03
	hsa-miR-330-3p	Y	qPCR (Fig. S3B)		
	hsa-miR-339-3p	Y	qPCR (Fig. S3B)		
	hsa-miR-376a	Y	qPCR (Fig. S3B)		
	hsa-miR-376c	Y	qPCR (Fig. S3B)		
	hsa-miR-377	N	miR-seq	0.73	1
	hsa-miR-423-3p	N*	miR-seq	0.52	0.029
	hsa-miR-486-5p	Y	miR-seq	-1.19	4.07E-04
	hsa-miR-495	N	miR-seq	-1.72	0.68
	hsa-miR-769-5p	N*	miR-seq	-0.83	1.48E-03
	hsa-miR-886-3p	N	miR-seq	NA	NA
HOXC6	hsa-miR-1	Y	qPCR (Fig. S3C)		
	hsa-miR-130a	Y	qPCR (Fig. S3C)		
	hsa-miR-133a	Y	qPCR (Fig. 2C)		
	hsa-miR-133b	N	miR-seq	NA	NA
	hsa-miR-143	Y	qPCR (Fig. S3C)		
	hsa-miR-145	Y	qPCR (Fig. 2C)		
	hsa-miR-205	Y	qPCR (Fig. 2C)		
	hsa-miR-221	Y	qPCR (Fig. 2C)		
	hsa-miR-27b	N	miR-seq	-0.24	0.93
	hsa-miR-31	N	miR-seq	-0.31	0.76
	hsa-miR-338-3p	N	qPCR (Fig. S3C)		
	hsa-miR-455	N	qPCR (Fig. S3C)		
	hsa-miR-495	N	miR-seq	-1.23	1
NKX2-2	hsa-miR-1	Y	qPCR (Fig. S3D)		
	has-miR-100	N	miR-seq	-0.37	0.57
	hsa-miR-125b	Y	miR-seq	3.41	9.98E-13

	hsa-miR-133a	Y	qPCR (Fig. 2C)		
	hsa-miR-133b	N	miR-seq	NA	NA
	hsa-miR-143	Y	qPCR (Fig. S3D)		
	hsa-miR-145	Y	qPCR (Fig. 2C)		
	hsa-miR-193b	Y	qPCR (Fig. S3D)		
	hsa-miR-205	Y	qPCR (Fig. 2C)		
	hsa-miR-221	Y	qPCR (Fig. 2C)		
	has-miR-27b	N*	miR-seq	-1.00	4.89E-03
	hsa-miR-365	Y	miR-seq	1.53	9.38E-03
	hsa-miR-455	Y	miR-seq	1.06	4.91E-03
	hsa-miR-886-3p	N	miR-seq	NA	NA
	hsa-miR-99a	Y	miR-seq	2.83	5.39E-13

1. NA indicates that no reads matching the microRNA were detected in miR-seq.

2. N* indicates that the TF does induce a significant expression change of the tested microRNA, but the direction of change is opposite of that established from the computational prediction.

3. MicroRNAs derived from the same precursor and displayed high correlation in expression are merged to simplify analyses, and to avoid inflating the validation rates. Specifically, the following four pairs of microRNAs are merged: has-miR-143 and has-miR-143* (cor = 0.84), has-miR-145 and has-miR-145* (cor = 0.93), has-miR-221 and has-miR-221* (cor = 0.76) and has-miR-455-5p and has-miR-455-3p (cor = 0.86).

Supplementary Table 2. Lists of microRNAs differentially expressed in metastatic prostate cancer and experimentally validated microRNAs co-regulated by AR, HOXC6 and NKX2-2.

	Experimentally validated microRNAs co-regulated by AR, HOXC6 and NKX2-2	MicroRNAs differentially expressed in metastatic prostate cancer	
		Taylor data set	Hart data set
Upregulated	hsa-miR-126 ↓[1]<br>hsa-miR-1285 ↑[2]<br>hsa-miR-1306<br>hsa-miR-1468<br>hsa-miR-182 ↑[3]<br>hsa-miR-192 ↑[4]<br>hsa-miR-224 ↓[5]<br>hsa-miR-30c ↓[6]<br>hsa-miR-320b ↑[7]<br>hsa-miR-330 ↓[8]<br>hsa-miR-33b ↓[9]<br>hsa-miR-486-5p ↑[10]<br>hsa-miR-5008<br>hsa-miR-561<br>hsa-miR-659<br>hsa-miR-671 ↑[11]<br>hsa-miR-873 ↓[12]	hsa-miR-135a*<br>hsa-miR-144<br>hsa-miR-183<br>hsa-miR-33b<br>hsa-miR-486-5p<br>hsa-miR-548c-3p<br>hsa-miR-550<br>hsa-miR-663	hsa-miR-106a<br>hsa-miR-106b<br>hsa-miR-10a<br>hsa-miR-141<br>hsa-miR-148a<br>hsa-miR-148b<br>hsa-miR-15a<br>hsa-miR-17<br>hsa-miR-186<br>hsa-miR-18a<br>hsa-miR-191<br>hsa-miR-195<br>hsa-miR-196b<br>hsa-miR-199b<br>hsa-miR-200a<br>hsa-miR-200b<br>hsa-miR-200c<br>hsa-miR-20a<br>hsa-miR-20b<br>hsa-miR-21<br>hsa-miR-23a<br>hsa-miR-25<br>hsa-miR-30d<br>hsa-miR-30e<br>hsa-miR-363<br>hsa-miR-375<br>hsa-miR-92a<br>hsa-miR-93
Downregulated	hsa-let-7c ↓[13]<br>hsa-miR-1 ↓[14]<br>hsa-miR-106a ↑[15]<br>hsa-miR-1180<br>hsa-miR-125b ↓[16]<br>hsa-miR-130a ↓[17]<br>hsa-miR-133a ↓[18]<br>hsa-miR-141 ↓[19]<br>hsa-miR-143 ↓[20]<br>hsa-miR-145 ↓[20]<br>hsa-miR-148b ↓[21]<br>hsa-miR-149 ↓[22]	hsa-miR-1<br>hsa-miR-100<br>hsa-miR-125b<br>hsa-miR-130a<br>hsa-miR-133a<br>hsa-miR-133b<br>hsa-miR-135a<br>hsa-miR-143<br>hsa-miR-145<br>hsa-miR-199b-5p<br>hsa-miR-204<br>hsa-miR-205	hsa-let-7b<br>hsa-let-7c<br>hsa-let-7e<br>hsa-miR-100<br>hsa-miR-130a<br>hsa-miR-143<br>hsa-miR-145<br>hsa-miR-205<br>hsa-miR-320a<br>hsa-miR-424<br>hsa-miR-451

	hsa-miR-17 ↓[23]	hsa-miR-221	
	hsa-miR-181a ↓[24]	hsa-miR-222	
	hsa-miR-181b ↓[24]	hsa-miR-224	
	hsa-miR-196b ↓[25]	hsa-miR-23b	
	hsa-miR-200c ↓[19]	hsa-miR-24-1*	
	hsa-miR-203a ↓[10]	hsa-miR-27b	
	hsa-miR-203b ↓[10]	hsa-miR-31	
	hsa-miR-20a ↓[26]	hsa-miR-886-3p	
	hsa-miR-20b ↓[27]	hsa-miR-99a	
	hsa-miR-205 ↓[17]		
	hsa-miR-221 ↓[28]		
	hsa-miR-301a ↑[29]		
	hsa-miR-301b ↑[30]		
	hsa-miR-3065		
	hsa-miR-342 ↓[31]		
	hsa-miR-34c ↓[13]		
	hsa-miR-363 ↓[10]		
	hsa-miR-421 ↓[32]		
	hsa-miR-454 ↓[10]		
	hsa-miR-548e		
	hsa-miR-675 ↓[33]		
	hsa-miR-7705		
	hsa-miR-9 ↓[32]		
	hsa-miR-92a ↑[34]		
	hsa-miR-99a ↓[35]		

1. Downregulated/upregulated refers to reduced/increased expression in metastatic prostate cancer comparing to normal prostate tissue for the Taylor and Hart data sets, and reduced/increased expression in RWPE-1 cells overexpressing AR, HOXC6 or NKX2-2 comparing to RWPE-1 control cells.

2. ↑indicates publications reporting the microRNA was upregulated in metastatic prostate cancer or promotes prostate cancer metastasis.

3. ↓indicates publications reporting the microRNA was downregulated in metastatic prostate cancer or inhibits prostate cancer metastasis.

4. ↑indicates publications reporting the microRNA promotes metastasis in non-prostate cancers.

5. ↓indicates publications reporting the microRNA inhibits metastasis in non-prostate cancers.

Supplementary Table 3. KEGG pathways enriched with genes targeted by microRNAs co-regulated by AR, HOXC6 and NKX2-2.

Term	Genes	FDR
Pathways in cancer	E2F1, HRAS, E2F3, PPARG, MITF, FOXO1, NFKB1, PTEN, MMP2, TGFB2, CCNE2, CDC42, FOS, RHOA, RALA, PIK3CA, FAS, MYC, TP53, RB1, CDK4, CCND1, HIF1A, VEGFA, MAPK9, FGFR3, XIAP, EGLN3, CDH1, BCL2L1, ITGB1, TCF7L1, IGF1R, KRAS, BCL2, RUNX1, PIK3R2, FN1, TGFBR1, MET, TGFBR2, SMAD4, STAT3, NRAS, CDKN1A, CDKN1B, ETS1, JAK1, MTOR, IKBKB, CRK	2.67E-21
Chronic myeloid leukemia	E2F1, HRAS, E2F3, NFKB1, BCL2L1, TGFB2, KRAS, PIK3CA, SHC1, RUNX1, MYC, PIK3R2, TGFBR1, TGFBR2, SMAD4, TP53, RB1, CDK4, PTPN11, NRAS, CDKN1A, CCND1, CDKN1B, IKBKB, CRK	1.61E-16
Pancreatic cancer	E2F1, E2F3, TGFBR1, TGFBR2, TP53, SMAD4, NFKB1, RB1, BCL2L1, CDK4, STAT3, TGFB2, CDC42, CCND1, KRAS, VEGFA, MAPK9, JAK1, RALA, PIK3CA, IKBKB, PIK3R2	2.44E-13
Prostate cancer	E2F1, HRAS, E2F3, CREB1, TP53, FOXO1, NFKB1, RB1, PTEN, TCF7L1, CCNE2, NRAS, IGF1R, CCND1, CDKN1A, KRAS, CDKN1B, BCL2, PIK3CA, MTOR, IKBKB, PIK3R2	3.58E-11
Bladder cancer	E2F1, HRAS, E2F3, FGFR3, TP53, CDH1, RB1, CDK4, MMP2, NRAS, CCND1, CDKN1A, KRAS, VEGFA, MYC	4.91E-09
Small cell lung cancer	E2F1, E2F3, XIAP, TP53, NFKB1, RB1, BCL2L1, CDK4, ITGB1, PTEN, CCNE2, CCND1, CDKN1B, BCL2, PIK3CA, IKBKB, MYC, FN1, PIK3R2	1.63E-08
Neurotrophin signaling pathway	HRAS, NTF3, TP53, NFKB1, FOXO3, IRS1, PTPN11, NTRK3, CDC42, NRAS, YWHAG, KRAS, MAPK14, BCL2, NTRK2, RHOA, MAPK9, PIK3CA, SHC1, IKBKB, CRK, PIK3R2	3.48E-08
Melanoma	E2F1, HRAS, E2F3, MET, MITF, TP53, CDH1, RB1, CDK4, PTEN, NRAS, IGF1R, CCND1, CDKN1A, KRAS, PIK3CA, PIK3R2	1.06E-07
Glioma	E2F1, HRAS, E2F3, TP53, RB1, CDK4, PTEN, NRAS, IGF1R, CCND1, CDKN1A, KRAS, PIK3CA, SHC1, MTOR, PIK3R2	1.82E-07
Cell cycle	E2F1, CDC7, E2F3, RBL2, RBL1, TP53, SMAD4, RB1, CDK4, ATM, WEE1, TGFB2, CCNE2, YWHAG, CCND1, CDKN1A, MAD2L1, CDKN1B, CCND2, TFDP2, MYC	3.11E-07
Acute myeloid leukemia	HRAS, PIM1, NFKB1, STAT3, TCF7L1, NRAS, CCND1, KRAS, PIK3CA, MTOR, RUNX1, IKBKB, MYC, PIK3R2	7.19E-06
Colorectal cancer	TGFBR1, MET, TGFBR2, TP53, SMAD4, TCF7L1, TGFB2, FOS, IGF1R, CCND1, KRAS, BCL2, MAPK9, PIK3CA, MYC, PIK3R2	1.31E-05
MAPK signaling pathway	HRAS, FGFR3, NFKB1, TGFB2, CDC42, FOS, KRAS, MAPT, FAS, MYC, PTPN7, NTF3, TGFBR1, NLK, TGFBR2, TP53, STK3, DUSP5, NRAS, MAPK14, NTRK2, MAPK9, STMN1, IKBKB, CRK, MAP3K12, DUSP6	3.58E-05
Renal cell carcinoma	CDC42, NRAS, HRAS, KRAS, HIF1A, ETS1, VEGFA, MET, EGLN3, PIK3CA, CRK, PIK3R2, TGFB2, PTPN11	8.00E-05
Endometrial cancer	NRAS, HRAS, CCND1, KRAS, TP53, PIK3CA, CDH1, FOXO3, MYC, PTEN, TCF7L1, PIK3R2	2.10E-04
Non-small cell lung cancer	E2F1, NRAS, HRAS, E2F3, CCND1, KRAS, TP53, PIK3CA, RB1, FOXO3, CDK4, PIK3R2	3.17E-04
T cell receptor signaling pathway	HRAS, CD8A, NFKB1, CDK4, IL10, CDC42, FOS, NRAS, KRAS, MAPK14, RHOA, ZAP70, PIK3CA, MAPK9, IKBKB, PIK3R2	4.24E-04
TGF-beta signaling pathway	RBL2, ROCK1, TGFBR1, TGFBR2, RBL1, SMAD4, BMPR2, TGFB2, ACVR2B, ID2, SP1, RHOA, BMP7, MYC	0.001126171

Focal adhesion	HRAS, FLT1, ROCK1, XIAP, MET, PTEN, ITGB1, CDC42, IGF1R, CCND1, CCND2, ITGA5, BCL2, VEGFA, RHOA, MAPK9, PIK3CA, SHC1, CRK, FN1, PIK3R2	0.00118287
Thyroid cancer	NRAS, HRAS, CCND1, KRAS, PPARG, TP53, CDH1, MYC, TCF7L1	0.001207037
Adherens junction	IGF1R, CDC42, NLK, TGFBR1, MET, TGFBR2, RHOA, SMAD4, CDH1, YES1, SNAI2, TCF7L1	0.012424939
p53 signaling pathway	CCNE2, CDKN1A, CCND1, CCND2, SERPINE1, TP53, FAS, PMAIP1, CDK4, PTEN, ATM	0.024373702
ErbB signaling pathway	NRAS, HRAS, CDKN1A, CDKN1B, KRAS, MAPK9, PIK3CA, SHC1, MTOR, CRK, MYC, PIK3R2	0.040544533

Supplementary Table 4. Gene expression data from FANTOM5 consortium demonstrates tissue-specific overexpression of AR, HOXC6 and NKX2-2.

Sample	AR (TPM cutoff for overexpression: 27.61)	HOXC6 (TPM cutoff for overexpression: 55.46)	NKX2-2 (TPM cutoff for overexpression: 15.37)
breast carcinoma cell line:MDA-MB-453.CNhs10736.10419-106C5	142.57	20.73	6.29
bone marrow stromal cell line:StromaNKtert.CNhs11931.10686-109F2	117.8	20.01	0
Hepatocyte, donor1.CNhs12340.11523-119I2	88.72	0	0
liver, adult, pool1.CNhs10624.10018-101C9	85	0	0
cervix, adult, pool1.CNhs10618.10013-101C4	77.02	2.61	0
seminal vesicle, adult.CNhs12851.10201-103F3	74.09	2.54	0
Hepatocyte, donor3.CNhs12626.11684-122I1	72.08	1.18	0
mature adipocyte, donor1.CNhs12558.12231-129F8	68.54	7.62	0
prostate, adult, pool1.CNhs10628.10022-101D4	61.54	4.32	0
mature adipocyte, donor3.CNhs12560.12233-129G1	59.12	14.09	0
mature adipocyte, donor4.CNhs12562.12234-129G2	57.38	9.83	0
Universal RNA - Human Normal Tissues Biochain, pool1.CNhs10612.10007-101B4	55.26	4.23	0
mature adipocyte, donor2.CNhs12559.12232-129F9	55.15	5.52	0
ductus deferens, adult.CNhs12846.10196-103E7	51.7	1.18	0
SABiosciences XpressRef Human Universal Total RNA, pool1.CNhs10610.10002-101A5	51.41	4.63	0.2
tridermal teratoma cell line:HGRT.CNhs11828.10694-109G1	48.27	0	0
acute myeloid leukemia (FAB M2) cell line:NKM-1.CNhs11864.10765-110E9	48.16	1.14	0
uterus, adult, pool1.CNhs11676.10100-102D1	46.81	23.47	0
epididymis, adult.CNhs12847.10197-103E8	42.81	29.01	0
pleomorphic hepatocellular carcinoma cell line:SNU-387.CNhs11933.10706-109H4	38.58	0	0
uterus, fetal, donor1.CNhs11763.10055-101H1	37.84	4.47	0
salivary acinar cells, donor3.CNhs12812.11773-123I9	35.95	0	0
ovary, adult, pool1.CNhs10626.10020-101D2	31.22	40.61	0
breast carcinoma cell line:MCF7.CNhs11943.10482-107A5	31	0.52	0
osteosarcoma cell line:143B/TK^(-)neo^(R).CNhs11279.10510-107D6	29	5.52	2.76
sacrococcigeal teratoma cell line:HTST.CNhs11829.10695-109G2	28.86	0	0
testis, adult, pool1.CNhs10632.10026-101D8	27.97	19.77	0
Preadipocyte - breast, donor1.CNhs11052.11467-119B9	1.48	192.03	0
Adipocyte - breast, donor1.CNhs11051.11376-118A8	8.73	164.78	0
Preadipocyte - omental, donor2.CNhs11902.11329-117E6	3.58	156.07	0
Preadipocyte - breast, donor2 (nuclear fraction).CNhs12584.14320-155D9	14.58	147.18	0
Fibroblast - skin normal, donor2.CNhs11914.11561-120D4	0.79	146.16	0
Fibroblast - skin normal, donor2 (nuclear fraction).CNhs12582.14302-155B9	1.69	145.99	0
Fibroblast - Lymphatic, donor1.CNhs11322.11506-119G3	4.55	136.52	0
Fibroblast - skin normal, donor1 (nuclear fraction).CNhs12403.14323-155E3	6.71	128.56	0
mesenchymal stem cells (adipose derived), adipogenic induction, 00hr00min, biol_rep2.CNhs13420.13230-141I8	3.09	114	0
Preadipocyte - breast, donor2.CNhs11971.11328-117E5	4.77	111.98	0
Mesenchymal Stem Cells - umbilical, donor1.CNhs11347.11539-120A9	1.63	110.16	0
Mesenchymal Stem Cells - adipose,	14.3	107.29	0

donor1.CNhs11345.11537-120A7			
Adipocyte - omental, donor2.CNhs12067.11474-119C7	5	107.08	0
Fibroblast - skin normal, donor1.CNhs11351.11553-120C5	2.8	105.89	0
Fibroblast - Lymphatic, donor3.CNhs12118.11667-122G2	2.88	102.05	0
mesenchymal stem cells (adipose derived), adipogenic induction, 00hr00min, biol_rep3.CNhs13421.13231-141I9	2.02	97.98	0
rhabdomyosarcoma cell line:RMS-YM.CNhs11269.10477-106I9	0.34	86.93	0
liposarcoma cell line:KMLS-1.CNhs11870.10782-110G8	1.53	85.2	0.17
Adipocyte - perirenal, donor1.CNhs12069.11476-119C9	6.1	83.75	0
Myoblast, donor3.CNhs11908.11398-118D3	9.32	80.75	0
Preadipocyte - perirenal, donor1.CNhs12065.11469-119C2	0	80.14	0
Adipocyte - breast, donor2.CNhs11969.11327-117E4	14.78	79.85	0
kidney, fetal, pool1.CNhs10652.10045-101F9	5.98	74.33	0
Fibroblast - skin dystrophia myotonica, donor1.CNhs11353.11556-120C8	2.56	71.75	0.42
Nucleus Pulposus Cell, donor1.CNhs10881.11252-116F1	7.37	69.07	0
mesenchymal precursor cell - adipose, donor2.CNhs12364.11748-123G2	6.07	68.47	0
synovial sarcoma cell line:HS-SY-II.CNhs11244.10441-106E9	0	67.84	6.94
Skeletal Muscle Cells, donor1.CNhs11083.11281-116I3	5.13	67.38	0
tenocyte, donor3.CNhs12641.11768-123I4	1.73	67.06	0
Fibroblast - skin spinal muscular atrophy, donor1 (nuclear fraction).CNhs12404.14326-155E6	3.02	66.14	0
Smooth Muscle Cells - Umbilical Vein, donor2.CNhs12569.11621-122B1	3.74	65.33	0
Fibroblast - skin spinal muscular atrophy, donor1.CNhs11074.11555-120C7	2.86	65.17	0
Anulus Pulposus Cell, donor1.CNhs10876.11248-116E6	1.6	64.37	0
Fibroblast - skin dystrophia myotonica, donor1 (nuclear fraction).CNhs12405.14329-155E9	5.56	64.23	0.18
Nucleus Pulposus Cell, donor2.CNhs12019.11409-118E5	4.5	61.51	0
Fibroblast - skin spinal muscular atrophy, donor3 (nuclear fraction).CNhs12398.14305-155C3	0.72	61.06	0
Smooth Muscle Cells - Umbilical Vein, donor1.CNhs12597.11541-120B2	2.72	60.53	0
Synoviocyte, donor3.CNhs12050.11440-118H9	0.99	59.6	0
tenocyte, donor1.CNhs12639.11763-123H8	0.74	58.77	0
spinal cord, fetal, donor1.CNhs11764.10056-101H2	4.22	58.76	19.86
Mesenchymal Stem Cells - umbilical, donor3.CNhs12127.11700-123A8	2.47	57.63	0
anaplastic squamous cell carcinoma cell line:RPMI 2650.CNhs11889.10805-111A4	0.42	57.42	0
mesenchymal precursor cell - adipose, donor3.CNhs12365.11749-123G3	2.77	57.14	0
neuroectodermal tumor cell line:FU-RPNT-1.CNhs11744.10637-108I7	0	56.54	0
Adipocyte - subcutaneous, donor3.CNhs12017.11408-118E4	15.93	56.41	0
neuroectodermal tumor cell line:FU-RPNT-2.CNhs11753.10663-109C6	0.49	36.31	151.33
neuroepithelioma cell line:SK-N-MC.CNhs11853.10728-110A8	7.26	10.61	107.27
spinal cord - adult, donor10196.CNhs13807.10181-103D1	3.74	2.36	75.97
optic nerve, donor1.CNhs13449.10277-104E7	0	0	57.12
osteosarcoma cell line:HS-Os-1.CNhs11290.10558-107I9	3.38	28.65	42.36
corpus callosum, adult, pool1.CNhs10649.10042-101F6	5.94	0.2	35.85

thalamus - adult, donor10196.CNhs13794.10168-103B6	4.15	0	34.83
substantia nigra, adult, donor10252.CNhs12318.10158-103A5	2.44	0	26.66
locus coeruleus - adult, donor10196.CNhs13808.10182-103D2	6.07	0	26.38
spinal cord, adult, donor10252.CNhs12227.10159-103A6	5.65	4.75	24.66
medulla oblongata, adult, pool1.CNhs10645.10038-101F2	5.65	1.55	22.04
medulla oblongata - adult, donor10196.CNhs13800.10174-103C3	2.87	0	20.07
pons, adult, pool1.CNhs10640.10033-101E6	3.06	3.33	19.92
mesothelioma cell line:ACC-MESO-4.CNhs11264.10494-107B8	0.42	2.79	19.51
thalamus, adult, donor10252.CNhs12314.10154-103A1	3.41	0	18.41
diencephalon, adult.CNhs12610.10193-103E4	3.51	0	18.05
medial temporal gyrus - adult, donor10196.CNhs13809.10183-103D3	0.85	0	17.45
globus pallidus - adult, donor10196.CNhs13801.10175-103C4	1.8	0	17.41
parietal lobe - adult, donor10196.CNhs13797.10171-103B9	0	0	17.18
globus pallidus, adult, donor10252.CNhs12319.10161-103A8	2.87	0	16.98
occipital lobe, adult, donor1.CNhs11787.10076-102A4	0.62	0	16.61
carcinoid cell line:SK-PN-DW.CNhs11846.10719-109I8	0	13.99	16.04

Samples overexpressing the indicated TF are highlighted for visual clarity.

Supplementary Table 5. AR, HOXC6 and NKX2-2 are not broadly associated with super-enhancers (SEs) in prostate cancer cell lines.

TF	Cell lines	Associated with SEs	Highest peak intensity of associated enhancers	Intensity cutoff for SEs	Percentile rank within all enhancers	GSE accession
AR	LnCAP	No	117.74	248.26	13%	GSE27824[36]
	PC3	No	36.19	69.38	25.9%	GSE57498[37]
	VCaP	**Yes**	152.68	95.72	0.6%	GSE55062[38]
HOXC6	LnCAP	No	189.12	248.26	6.3%	GSE27824
	PC3	No	14.89	69.38	63.7%	GSE57498
	VCaP	No	91.79	95.72	3.1%	GSE55062
NKX2-2	LnCAP	No	4.16	248.26	97.7%	GSE27824
	PC3	No	63.94	69.38	7.9%	GSE57498
	VCaP	No	66.18	95.72	7%	GSE55062

Supplementary Table 6. Primer sequences for qRT-PCR.

U6 forward	CTCGCTTCGGCAGCACA
U6 reverse	AACGCTTCACGAATTTGCGT
Unified reverse primer	CCAGTGCAGGGTCCGAGGTA
hsa-miR-1-3p forward	TGGAATGTAAAGAAGTATGTAT
hsa-miR-1237 forward	TCCTTCTGCTCCGTCCCCCAG
hsa-miR-130a forward	CAGTGCAATGTTAAAAGGGCAT
hsa-miR-133a-3p forward	TTTGGTCCCCTTCAACCAGCTG
hsa-miR-143-3p forward	TGAGATGAAGCACTGTAGCTC
hsa-miR-145-5p forward	GTCCAGTTTTCCCAGGAATCC
hsa-miR-17-5p forward	CACGCACAACGTGCTTACAGTGC
hsa-miR-193b-3p forward	AACTGGCCCTCAAAGTCCCGCT
hsa-miR-199a forward	CCCAGGGGGCAGACGACCGGGGC
hsa-miR-199b-5p forward	CCCAGGGGGCAGACGACCGGGGC
hsa-miR-200c-3p forward	GCCCGCTAATACTGCCGGGTAAT
hsa-miR-205-5p forward	TCCACCGGAGTCTGGTCGTAT
hsa-miR-221-3p forward	TTGTCTGCTGGGTTTCGTCG
hsa-miR-330-3p forward	CAAAGCACACGGCCTGCAGAGA
hsa-miR-338-3p forward	TCCAGCATCAGTGATTTTGTTG
hsa-miR-339-3p forward	TGAGCGCCTCGACGACAGAGCCG
hsa-miR-376a-5p forward	TAGATTCTCCTTCTATGAGTA
hsa-miR-376c-5p forward	GGTGGATATTCCTTCTATGTT
hsa-miR-455-5p forward	TATGTGCCTTTGGACTACATCG
AR forward	CCAGGGACCATGTTTTGCC
AR reverse	CGAAGACGACAAGATGGACAA

Supplementary Table 7. Sequences for siRNAs and microRNA mimics.

AR-homo 1 sense	GCAGAAAUGAUUGCACUAUTT
AR-homo 1 antisense	AUAGUGCAAUCAUUUCUGCTT
AR-homo 2 sense,	GACAGUGUCACACAUUGAATT
AR-homo 2 antisense	UUCAAUGUGUGACACUGUCTT
HOXC6-homo 1 sense	CCUCAAUUCCACCGCCUAUTT
HOXC6-homo 1 antisense	AUAGGCGGUGGAAUUGAGGTT
HOXC6-homo 2 sense	GAGAAUGUCGUGUUCAGUUTT
HOXC6-homo 2 antisense	AACUGAACACGACAUUCUCTT
NKX2-2-homo 1 sense	CGGUCAAGGACAUCUUAGATT
NKX2-2-homo 1 antisense	UCUAAGAUGUCCUUGACCGTT
NKX2-2-homo 2 sense	GCAGCACAUGCAGUACAACTT
NKX2-2-homo 2 antisense	GUUGUACUGCAUGUGCUGCTT
LSD1-homo sense	CGAAGGUAGAGUACAGAGATT
LSD1-homo antisense	UCUCUGUACUCUACCUUCGTT
has-miR-133a-3p mimics	UUUGGUCCCCUUCAACCAGCUG
has-miR-145-5p mimics	GUCCAGUUUUCCCAGGAAUCCCU
has-miR-205-5p mimics	UCCUUCAUUCCACCGGAGUCUG

Supplementary References

1. Tai HC, *et al.* Osteoblast-derived WNT-induced secreted protein 1 increases VCAM-1 expression and enhances prostate cancer metastasis by down-regulating miR-126. *Oncotarget* **5**, 7589-7598 (2014).

2. Chen ZH, *et al.* A panel of five circulating microRNAs as potential biomarkers for prostate cancer. *Prostate* **72**, 1443-1452 (2012).

3. Rasheed SA, Teo CR, Beillard EJ, Voorhoeve PM, Casey PJ. MicroRNA-182 and microRNA-200a control G-protein subunit alpha-13 (GNA13) expression and cell invasion synergistically in prostate cancer cells. *The Journal of biological chemistry* **288**, 7986-7995 (2013).

4. Valencia K, *et al.* miRNA cargo within exosome-like vesicle transfer influences metastatic bone colonization. *Molecular oncology* **8**, 689-703 (2014).

5. Lin ZY, *et al.* MicroRNA-224 inhibits progression of human prostate cancer by downregulating TRIB1. *International journal of cancer* **135**, 541-550 (2014).

6. Kao CJ, *et al.* miR-30 as a tumor suppressor connects EGF/Src signal to ERG and EMT. *Oncogene* **33**, 2495-2503 (2014).

7. Zhou J, *et al.* MicroRNA-320b promotes colorectal cancer proliferation and invasion by competing with its homologous microRNA-320a. *Cancer letters* **356**, 669-675 (2015).

8. Mao Y, *et al.* microRNA-330 inhibits cell motility by downregulating Sp1 in prostate cancer cells. *Oncology reports* **30**, 327-333 (2013).

9. Lin Y, *et al.* MicroRNA-33b Inhibits Breast Cancer Metastasis by Targeting HMGA2, SALL4 and Twist1. *Sci Rep* **5**, 9995 (2015).

10. Watahiki A, *et al.* MicroRNAs associated with metastatic prostate cancer. *PloS one* **6**, e24950 (2011).

11. Tan X, *et al.* miR-671-5p promotes epithelial-to-mesenchymal transition by downregulating FOXM1 expression in breast cancer. *Cancer Research* **75**, (2015).

12. Wang RJ, *et al.* MicroRNA-873 (miRNA-873) inhibits glioblastoma tumorigenesis and metastasis by suppressing the expression of IGF2BP1. *The Journal of biological chemistry* **290**, 8938-8948 (2015).

13. Ngalame NN, Tokar EJ, Person RJ, Xu Y, Waalkes MP. Aberrant microRNA expression likely controls RAS oncogene activation during malignant transformation of human prostate epithelial and stem cells by arsenic. *Toxicological sciences : an official journal of the Society of Toxicology* **138**, 268-277 (2014).

14. Chang YS, Chen WY, Yin JJ, Sheppard-Tillman H, Huang J, Liu YN. EGF Receptor Promotes Prostate Cancer Bone Metastasis by Downregulating miR-1 and Activating TWIST1. *Cancer Res* **75**, 3077-3086 (2015).

15. Feng B, *et al.* Colorectal cancer migration and invasion initiated by microRNA-106a. *PloS one* **7**, e43452 (2012).

16. Wang M, *et al.* Loss of miR-100 enhances migration, invasion, epithelial-mesenchymal transition and stemness properties in prostate cancer cells through targeting Argonaute 2. *International journal of oncology* **45**, 362-372 (2014).

17. Boll K, *et al.* MiR-130a, miR-203 and miR-205 jointly repress key oncogenic pathways and are downregulated in prostate carcinoma. *Oncogene* **32**, 277-285 (2013).

18. Kojima S, *et al.* Tumour suppressors miR-1 and miR-133a target the oncogenic function of purine nucleoside phosphorylase (PNP) in prostate cancer. *British journal of cancer* **106**, 405-413 (2012).

19. Banyard J, *et al.* Regulation of epithelial plasticity by miR-424 and miR-200 in a new prostate cancer metastasis model. *Sci Rep* **3**, 3151 (2013).

20. Peng X, *et al.* Identification of miRs-143 and -145 that is associated with bone metastasis of prostate cancer and involved in the regulation of EMT. *PloS one* **6**, e20341 (2011).

21. Cimino D, *et al.* miR148b is a major coordinator of breast cancer progression in a relapse-associated microRNA signature by targeting ITGA5, ROCK1, PIK3CA, NRAS, and CSF1. *FASEB journal : official publication of the Federation of American Societies for Experimental Biology* **27**, 1223-1235 (2013).

22. Schaefer A, *et al.* Diagnostic and prognostic implications of microRNA profiling in prostate carcinoma. *International journal of cancer* **126**, 1166-1176 (2010).

23. Zhang X, Ladd A, Dragoescu E, Budd WT, Ware JL, Zehner ZE. MicroRNA-17-3p is a prostate tumor suppressor in vitro and in vivo, and is decreased in high grade prostate tumors analyzed by laser capture microdissection. *Clin Exp Metastasis* **26**, 965-979 (2009).

24. Cao Q, *et al.* Coordinated regulation of polycomb group complexes through microRNAs in cancer. *Cancer cell* **20**, 187-199 (2011).

25. Li Y, *et al.* Ratio of miR-196s to HOXC8 messenger RNA correlates with breast cancer cell migration and metastasis. *Cancer Res* **70**, 7894-7904 (2010).

26. Yan H, *et al.* MicroRNA-20a overexpression inhibited proliferation and metastasis of pancreatic carcinoma cells. *Human gene therapy* **21**, 1723-1734 (2010).

27. Saleiban A, Faxalv L, Claesson K, Jonsson JI, Osman A. miR-20b regulates expression of proteinase-activated receptor-1 (PAR-1) thrombin receptor in melanoma cells. *Pigment cell & melanoma research* **27**, 431-441 (2014).

28. Sun T, *et al.* MiR-221 promotes the development of androgen independence in prostate cancer cells via downregulation of HECTD2 and RAB1A. *Oncogene* **33**, 2790-2800 (2014).

29. Nam RK, *et al.* MiR-301a regulates E-cadherin expression and is predictive of prostate cancer recurrence. *Prostate*, (2016).

30. Funamizu N, Lacy CR, Parpart ST, Takai A, Hiyoshi Y, Yanaga K. MicroRNA-301b promotes cell invasiveness through targeting TP63 in pancreatic carcinoma cells. *International journal of oncology* **44**, 725-734 (2014).

31. Li X, *et al.* MicroRNA-185 and 342 inhibit tumorigenicity and induce apoptosis through blockade of the SREBP metabolic pathway in prostate cancer cells. *PloS one* **8**, e70987 (2013).

32. Ostling P, *et al.* Systematic analysis of microRNAs targeting the androgen receptor in prostate cancer cells. *Cancer Res* **71**, 1956-1967 (2011).

33. Zhu M, *et al.* lncRNA H19/miR-675 axis represses prostate cancer metastasis by targeting TGFBI. *The FEBS journal* **281**, 3766-3775 (2014).

34. Li Y, Kong D, Ahmad A, Bao B, Sarkar FH. Targeting bone remodeling by isoflavone and 3,3'-diindolylmethane in the context of prostate cancer bone metastasis. *PloS one* **7**, e33011 (2012).

35. Sun D, *et al.* miR-99 family of MicroRNAs suppresses the expression of prostate-specific

antigen and prostate cancer cell proliferation. *Cancer Res* **71**, 1313-1324 (2011).

36. Wang D*, et al.* Reprogramming transcription by distinct classes of enhancers functionally defined by eRNA. *Nature* **474**, 390-394 (2011).

37. Taberlay PC, Statham AL, Kelly TK, Clark SJ, Jones PA. Reconfiguration of nucleosome-depleted regions at distal regulatory elements accompanies DNA methylation of enhancers and insulators in cancer. *Genome Res* **24**, 1421-1432 (2014).

38. Asangani IA*, et al.* Therapeutic targeting of BET bromodomain proteins in castration-resistant prostate cancer. *Nature* **510**, 278-282 (2014).
